# Can the CANKADO online application improve quality of life monitoring via the endometriosis health profile-30 in endometriosis patients: A randomized cohort study on acceptance, usability, and correlations with demographics and media usage

**DOI:** 10.1186/s12905-025-03736-w

**Published:** 2025-04-28

**Authors:** Lucia Ehmann, Maresa Jäger, Lina Folger, Timo Schinköthe, Susanne Beyer, Lennard Schröder, Sven Mahner, Thomas Kolben

**Affiliations:** 1 Department of Obstetrics and Gynecology, University Hospital Munich, Ludwig-Maximilians University, Munich, Germany; 2 Gyn Munich, Gynecologic Surgery, Heidemannstr. 5b, 80939 Munich, Germany; 3 CANKADO GmbH, Munich, Germany; 4 Research Center Smart Digital Health, University of the Bundeswehr, Munich, Germany

**Keywords:** Endometriosis, Health-related quality of life, EHP-30, Electronic patient-reported outcome measurement, CANKADO

## Abstract

**Background:**

The global increase in interest in endometriosis highlights the importance of further investigations concerning this so-called benign gynecological disease. Owing to their severe presentation of symptoms, patients suffer from an enormous impact on their health-related quality of life (HRQoL). While the paper-based assessment of quality of life via, e.g., the “Endometriosis Health Profile-30 questionnaire (EHP-30)” seems to be largely accepted and implemented, the electronic measurement of this patient-reported outcome (ePRO) is still rarely applied. This study aimed to analyze the acceptance and usability of electronic assessments of HRQoL in endometriosis patients via the online platform CANKADO.

**Methods:**

The study was conducted at the Department of Gynecology and Obstetrics of LMU Munich between January 2022 and February 2023. Sixty conservatively treated patients with endometriosis were recruited for the randomized cohort study, followed by randomization due to their planned interrogation modality (n paper-based = 23, n online-based = 17). Afterwards, a HRQoL assessment via the EHP-30 questionnaire was performed. An evaluation of the interrogation modalities was performed at 0, 6 and 12 months. The metric or categorical variables were compared via Fisher’s exact test or the Mann‒Whitney U test. Correlation analysis was performed by calculating the Kendall Tau coefficient or Eta coefficient.

**Results:**

Forty patients completed evaluation forms at T0 (0 months), with *n* = 23 evaluating the paper-based interrogation modality and *n* = 17 evaluating the online version. At all the time of assessment, more than 80% of the patients showed a positive response to routinely performed ePRO measurements in the clinical context, expecting simplified communication, faster diagnosis, and therapeutic improvement. The online modality was rated more suitably (T0: 72.7% vs. 76.5%; T3: 60.0% vs. 90.0%), less complex (T0: 59.1% vs. 76.5%; T3: 80.0% vs. 70.0%), and less laborious (T0: 72.7% vs. 70.6%; T3: 80% each). Completion time over ten minutes was significantly correlated with low coping ability (*r* = 0.530; *p* = 0.029), lower clarity (*r* = 0.530; *p* = 0.029) and greater effort (*r* = 0.593; *p* = 0.012).

**Conclusions:**

The findings indicate high acceptance and usability of regularly performed ePRO assessments in patients with endometriosis via the online tool CANKADO.

## Introduction

According to previous research, an increase in the prevalence of endometriosis, as well as the number of patients with endometriosis, was recently reported in Germany, illustrating the importance of further investigations in this field [1–3]. Owing to the severe and complex appearance of symptoms, the physical, mental and social health-related quality of life of affected individuals is strongly impaired [4]. While the paper-based assessment of the patient-reported outcome ‘quality of life’ via the endometriosis-specific questionnaire Endometriosis Health Profile 30 seems to be largely accepted and implemented, electronic measurement in a clinical setting is still rarely applied [5, 6]. Patient-reported outcomes (PROs) are defined as self-assessed statements made by patients about their health status without any interpretation by medical staff. They can be determined as absolute values, such as the intensity of symptoms and stage of disease, while changes in parameters can also be measured. By means of this subjective information, the outcome of therapeutic interventions can be evaluated and adjusted [7]. While the classical approach includes paper-based PRO assessment, the electronic measurement of so-called ePROs, with the help of online-based interrogation modalities, has received greater focus in recent research. Previous studies that applied electronic PRO assessment in oncological patients reported a decrease in hospitalization and emergencies, whereas an increase in therapy duration and overall survival was observed [8, 9]. Another great advantage was investigated in reference to simplified communication and thus improved doctor‒patient relationships, as well as improved therapy adherence in patients with breast cancer [10]. With the increased development of medical health applications in recent years, a combination of ePRO assessments, integrated into medical product-approved online platforms, could be the next logical step. Endometriosis-specific applications, such as the Endo app developed in Germany, focus on increasing self-management via digital diaries, education modules and other disease-related information while already digitally integrating a HRQoL assessment [11]. In contrast, the absence of an adequate ePRO assessment for both patients and practitioners, with the aims of simplified doctor–patient–communication, pre- and postinterventional quality-of-life assessments and subsequent improvements in diagnostic and therapeutic regimens, is still prevalent. A previous investigation of the online application CANKADO provided the first results concerning improved therapy monitoring and adherence in patients with breast cancer [10, 12, 13]. This study was conducted to assess the acceptance and usability of the CANKADO for HRQoL measurement in patients with endometriosis to allow further research regarding the implementation of eHealth applications in the clinical setting.

## Methods

### Participants and recruitment

Patient recruitment for the randomized cohort study started in January 2022, ended in February 2023, and was conducted in the endometriosis-specific consultation of the Department of Gynecology and Obstetrics at LMU Munich. Before consultation with a gynecologist specializing in the diagnosis and treatment of endometriosis, patients received an information sheet explaining the aim, design and duration of the study. Thus, informed consent could be obtained if eligibility requirements were fulfilled. The inclusion criteria for the study included (1) clinically or previously surgically diagnosed endometriosis. Clinical diagnosis included detailed anamnesis, vaginal inspection with a speculum, bimanual and rectovaginal palpation, transvaginal sonography and, if necessary, rectal sonography. Furthermore, (2) the presence of endometriosis-specific symptoms, such as cyclical pelvic pain, dysmenorrhea, dyspareunia, dysuria or dyschezia, necessitating a surgical or conservative treatment approach; (3) being of legal age or providing parental consent; (4) being a German native speaker or having sufficient German language comprehension skills for completing the EHP-30 questionnaire; and (5) providing informed consent. Owing to our study location at a German university clinic and the implementation of an online application with a German user interface, the German version of the EHP-30 questionnaire was chosen as part of our study design. The exclusion criteria included (1) insufficient clinical or surgical evidence for the diagnosis of endometriosis, (2) the absence of symptoms impeding the need for any treatment and (3) a desire for pregnancy, thereby limiting acute treatment opportunities. (4) The absence of an informed consent declaration led to the exclusion of the study.

The 60 endometriosis patients included in the current study were part of a superordinate study with an overall population of 90 patients. After a first-stage randomization, owing to their therapeutic regime, which was determined during the gynecological consultation, these 60 patients were randomized into the conservatively treated therapy arm. Conservative treatment included hormone therapy, analgesics, and other nonsurgical approaches. Thirty patients in this superordinate study were randomized into a surgically treated therapy arm, in which different outcomes concerning interrogation frequency were investigated. For this reason, the following results refer exclusively to the conservative therapy arm.

Within the conservative therapy group, a second-stage block randomization into a paper-based and online-based study arm, in reference to the planned EHP-30 interrogation modality, was performed. Therefore, patients had to elect a previously prepared blank envelope containing a notification with their chosen interrogation modality and thus a future study arm. Several envelopes were prepared in advance, each containing different paper-based notifications labeled with either the paper-based or online-based study group. After envelope preparation and closure, neither the practitioner nor the patients knew the content of the prepared blank envelopes, leading to a double-blind randomization setting. For the paper-based study arm, the classic paper version of the EHP-30 questionnaire was handed to the patients. The online-based EHP-30 questionnaire had previously been integrated into the online platform CANKADO and thus was completed electronically. Additionally, evaluation sheets to rate the acceptance and usability of the two interrogation modalities at 0, 6 and 12 months were handed to the patients [14]. Within the evaluation sheets, patients were able to rate different parameters (e.g., scope range, clarity, complexity of their interrogation modality) on scales ranging from 1 to 10. For descriptive interpretation, numbers from 1 to 5 and 6–10 were later summarized into two response categories, leading to binary answers, as shown in Table 1. While the variable “complexity” of the interrogation modality referred to difficulties in comprehension, “laboriousness” referred to interrogation modality-related exhaustion. Completion time was estimated by patients after completing either the paper- or online-based questionnaire.

**Table 1:** Descriptive analysis of the evaluation data at T0

Category	Paper	Online	Exact Fisher’s test (two-sided)
**Scope range**	*n* = 23	*n* = 17	*p* = 0.705
Too few questions	4 (17.4%)	1 (5.9%)	
Adequate	15 (65.2%)	12 (70.6%)	
Too many questions	4 (17.4%)	4 (23.5%)	
**Completion time [minutes]**	*n* = 22	*n* = 17	***p*** ** = 0.017** ^a/**^
Mean	7.18	10.35	
SD	5.89	5.69	
Range	29 [1–30]	21 [4–25]	
**Coping**	*n* = 23	*n* = 17	*p* = 1.000
Good	18 (78.3%)	14 (82.4%)	
Poor	5 (21.7%)	3 (17.6%)	
**Clarity**	*n* = 23	*n* = 17	*p* = 1.000
Good	20 (87.0%)	14 (82.4%)	
Poor	3 (13.0%)	3 (17.6%)	
**Effort**	*n* = 23	*n* = 17	*p* = 0.702
Low	19 (82.6%)	13 (76.5%)	
High	4 (17.4%)	4 (23.5%)	
**In favor of routine interrogation**	*n* = 23	*n* = 17	*p* = 0.425
Yes	23 (100%)	16 (94.1%)	
No	0	1 (5.9%)	
**Counter reason**	*n* = 23	*n* = 17	
No concern	18 (78.3%)	9 (52.9%)	*p* = 0.171
Data privacy	4 (17.4%)	6 (35.3%)	*p* = 0.274
General additional burden	2 (8.7%)	2 (11.8%)	*p* = 1.000
Additional health burden	0	2 (11.8%)	*p* = 0.174
Lack of technical knowledge	0	1 (5.9%)	*p* = 0.425
**Suitability**	*n* = 22	*n* = 17	*p* = 1.000
Good	16 (72.7%)	13 (76.5%)	
Poor	6 (27.3%)	4 (23.5%)	
**Complexity**	*n* = 22	*n* = 17	*p* = 0.318
Low	13 (59.1%)	13 (76.5%)	
High	9 (40.9%)	4 (23.5%)	
**Laboriousness**	*n* = 22	*n* = 17	*p* = 1.000
Low	16 (72.7%)	12 (70.6%)	
High	6 (27.3%)	5 (29.4%)	
**Care improvement**	*n* = 23	*n* = 17	*p* = 0.499
Yes	21 (91.3%)	17 (100%)	
No	2 (8.7%)	0	
**Reasons for improvement**	*n* = 23	*n* = 17	
Simplified communication	19 (95.0%)	13 (76.5%)	*p* = 0.702
Patient-centeredness	9 (45.0%)	7 (41.2%)	*p* = 1.000
Fewer emergencies	2 (10.0%)	5 (29.4%)	*p* = 0.113
Faster diagnostics	13 (65.0%)	11 (64.7%)	*p* = 0.747
Improved therapeutic concepts	13 (65.0%)	16 (94.1%)	***p*** ** = 0.012** ^**^

^a^Mann-Whitney-U Test, **significant (α = 5%)

**Fig. 1:**
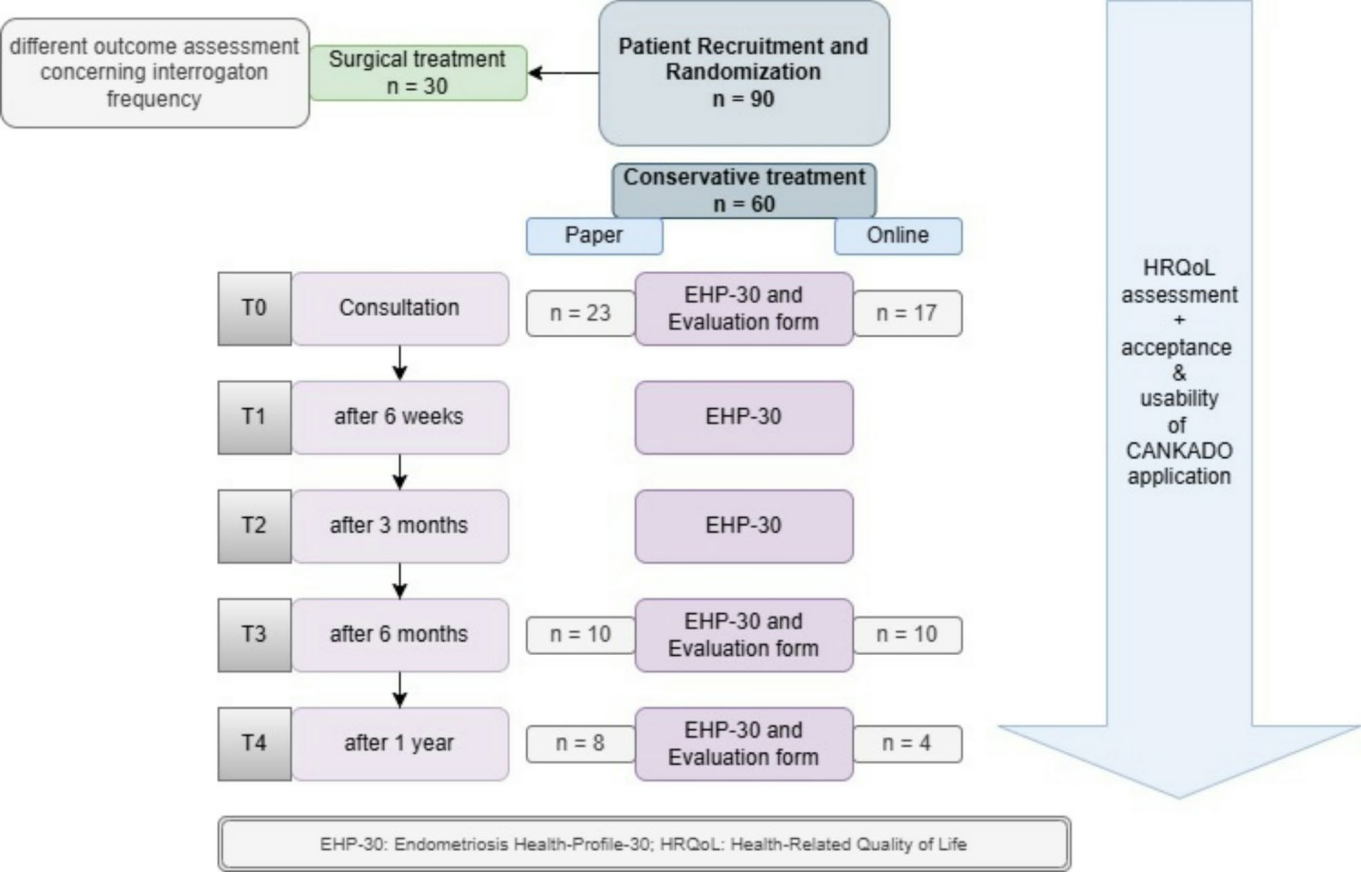
Flow diagram of the study design

### Measures

#### Health-related quality of life

For the assessment of the Patient-Reported Outcome ‘Health Related Quality of Life’, the Endometriosis Health Profile-30 (EHP-30) was used. This widely accepted and implemented endometriosis-specific questionnaire was developed by Jones et al. at the University of Oxford [5]. It contains a core questionnaire with 30 items referring to general gynecological symptoms and a modular questionnaire with 23 items, more specifically aimed at symptoms of endometriosis. The items of the core questionnaire are distributed in the following five subscales: pain, control and powerlessness, emotional well-being, social support, and self-image. The other six disease-specific subscales are work, sexual intercourse, relationships with children, medical profession, treatment, and infertility. Answers can be rated on a 5-point Likert scale (0 = never to 4 = always). By adding the scale numbers and then dividing the total number of scores and multiplying by 100, a score from 0 to 100 could be calculated, with a higher score indicating a reduced HRQoL. In our study, we used the German version of the EHP-30. The health-related quality of life in the conservative therapy group was then assessed at the first consultation (T0) and after six weeks (T1), three months (T2), six months (T3) and one year (T4). Reminder e-mails were sent to patients for postal return to the EHP-30 questionnaire. The patients in the online-based study arm received a reminder e-mail and additional automatic notifications via the online application.

#### Evaluation of interrogation modality

To evaluate their assigned interrogation mode, patients were handed specially created paper-based evaluation forms. While both types of evaluation forms consisted of the subsections ‘Conception of the questionnaire,’ ‘Media usage’ and ‘Demographics’, the version for the online-based group additionally contained the part ‘Handling of the digital questionnaire’ with the subscale’s operation, navigation, section change, section completion and usability. Patients were asked to rate each subcategory on a scale from 1 to 10. With respect to the categories “Counter reasons” and “Reasons for improvement”, patients were able to provide multiple answers. The metric variable completion time was estimated by patients after completion of their elected interrogation modality. All necessary documents were handed to the patients at the first consultation together with stamped envelopes for postal return during the 12-month follow-up. Reminder e-mails for the evaluation form were sent after six months (T3) and one year (T4).

#### CANKADO

The online application CANKADO is a medical device developed in Germany for capturing and interpreting active and passive PRO. In our study, the EHP-30 questionnaire was included in the online application to enable electronic collection of the active PRO “Health-related Quality of Life”. For passive PRO assessment, self-tracker or wearable data can be collected, as shown in earlier studies [15, 16]. Furthermore, the PRO-React system can be activated to manage side effects, e.g., when patients are receiving systemic therapy [12, 17], providing advice for further consultation by a specialized practitioner in case of exacerbation of symptoms. Patients are able to add their current medication and ensure regular medication intake via reminder tools. Additionally, health status and additional anamnestic information can be specified within a diary section. An included visual analog scale for estimation of current health status and pain allows more detailed symptom monitoring and gives patients and practitioners an opportunity to observe the development of symptoms. Healthcare professionals can use the system via the web portal or through direct integration via the hospital information system. Patients were free to use the application on their smartphone, tablet, or computer.

Earlier studies have already shown promising results of the use of this method in reference to therapy monitoring and thereby improvement in therapy adherence in breast cancer patients. The main objective was to improve doctor–patient communication in the outpatient clinical setting [10]. After the randomization process, patients in the online-based study arm received a welcome sheet with a QR code for registration in the online application. After registration, they were offered to change their username and create a private password to ensure the security of their personal data. Only the medical professionals of our investigation team and certain IT members of CANKADO were permitted insight into the patients yet previously pseudonymized data. These measures ensured data security for our participants.

### Statistical analysis

The statistical analysis of the collected data was performed with IBM SPSS Statistics, version 28. Owing to the small sample size, no test for distribution of normality was performed; thus, the data were declared nonparametric. For the description of demographics and results of the evaluation forms, a descriptive statistical analysis was conducted for both study arms. Metric variables are presented as the mean values, standard deviations, and ranges, and categorical variables are presented as absolute numbers and percentages. For the comparison of nonparametric, unpaired, binary group variables, an exact Fisher’s test was applied. In the case of nonparametric, unpaired, metric group variables, a Mann‒Whitney U test was conducted. Furthermore, a correlation analysis between the evaluation results and variables such as age, completion time, media usage and IT skills was performed. For the correlation between the metric and ordinally scaled variables, the Eta coefficient was calculated. When two ordinally scaled variables were compared, the Kendall-Tau c coefficient was applied because of the bivariate distribution and small sample size (< 50). A significance value of 5% was applied, and a positive correlation was interpreted when *r* > 0.3 [18].

## Results

### Descriptive analysis

A total of 60 patients in the conservative therapy group were enrolled in the study. Fifty-six patients completed evaluation forms, of whom 23 patients (41.1%) evaluated the paper-based modality and 17 patients (30.4%) evaluated the online version for a subsequent interpersonal comparison. Owing to an additionally planned intrapersonal comparison, another 16 patients completed both the paper-based and the online versions of the EHP-30 questionnaire. The focus of this study was the investigation of an interpersonal comparison. At T3, a total of 20 patients returned evaluation forms, *n* = 10 (17.9%) evaluating the paper-based questionnaire and *n* = 10 (17.9%) the online interrogation modality. Given the low rate of return, not enough data for appropriate statistical analysis were obtained at T4 (after one year).

The demographics of both groups were homogeneous for all the variables assessed, as shown in Table 2.

**Table 2:** Sociodemographic characteristics of the Conservative therapeutic study group at T0

	All	Paper	Online	Exact Fisher’s test (two-sided)
**Conservative therapy**	*n* = 60	*n* = 24	*n* = 36	
**Evaluation forms**	*n* = 56	*n* = 23	*n* = 33	
**Age** [years]	*n* = 56	*n* = 23	*n* = 33	*p* = 0.117^a^
Mean	29.52	27.48	30.94	
SD	7.89	6.19	8.7	
Range	35 [17–52]	24 [18–42]	35 [17–52]	
**Marital status**	*n* = 51^b^	*n* = 22	*n* = 29^b^	
Married	9 (17.6%)	5 (22.7%)	4 (13.8%)	*p* = 0.464
Partnership	20 (39.2%)	10 (45.5%)	10 (34.5%)	*p* = 0.395
Single	18 (35.3%)	7 (31.8%)	11 (37.9%)	*p* = 1.000
Divorced	4 (7.8%)	0	4 (13.8%)	*p* = 0.132
**Education**	*n* = 49^b^	*n* = 20	*n* = 29^b^	
Secondary school	18 (36.7%)	8 (40.0%)	10 (34.5%)	*p* = 0.777
Technical college	9 (18.4%)	3 (15.0%)	6 (20.7%)	*p* = 0.720
Abitur	22 (44.9%)	9 (45.0%)	13 (44.8%)	*p* = 1.000
**Employment**	*n* = 51^b^	*n* = 22	*n* = 29^b^	
Full-time	25 (49.0%)	12 (54.5%)	13 (44.8%)	*p* = 0.575
Part-time	14 (27.5%)	6 (27.3%)	8 (27.6%)	*p* = 1.000
Sick leave	3 (5.9%)	1 (4.5%)	2 (6.9%)	*p* = 1.000
Unemployed	9 (17.6%)	3 (13.6%)	6 (20.7)	*p* = 0.717
**Children**	*n* = 53	*n* = 22	*n* = 31	
Yes	8 (15.1%)	2 (9.1%)	6 (19.4%)	*p* = 0.666
No	45 (84.9%)	20 (90.9%)	25 (80.6%)	
**Number of children**	*n* = 54	*n* = 22	*n* = 32	*p* = 0.409^a^
Mean	0.3	0.27	0.31	
SD	0.79	0.88	0.74	
Range	3 [0–3]	3 [0–3]	3 [0–3]	
**Interpersonal Comparison**	*n* = 40	*n* = 23	*n* = 17	
**Media Usage**	*n* = 40	*n* = 23	*n* = 17	
Smartphone	39^c^ (100%)	22^c^ (100%)	17 (100%)	const.
Computer	28 (70.0%)	18 (78.3%)	10 (58.8%)	*p* = 0.296
Internet	51 (94.4%)	21 (91.2%)	17 (100%)	*p* = 0.499
Mobile Phone	18 (45.0%)	12 (52.2%)	6 (35.3%)	*p* = 0.348
Tablet	12^c^ (30.8%)	6^c^ (26.1%)	6 (35.3%)	*p* = 0.730
**IT skills**	*n* = 40	*n* = 23	*n* = 17	***p*** ** = 0.026** ^**^
Low	4 (10.0%)	0	4 (23.5%)	
Good to Professional	36 (90.0%)	23 (100%)	13 (76.5%)	

^a^Mann-Whitney-U Test; ^b^adjusted to multiple response of one individual, ^c^adjusted to one missing value, SD: standard deviation, const.: constant variable, **significant (α = 5%)

In reference to the results of the ‘Media usage’ section, a significant difference was observed in the category of IT skills (*p* = 0.026). The results of the descriptive analysis concerning the subsection ‘Conception of the questionnaire’ at T0 are shown in Table 1.

Compared with the paper-based version, the online modality was rated as more suitable (T0: 72.7% vs. 76.5%), less complex (T0: 59.1% vs. 76.5%), and less laborious (T0: 72.7% vs. 70.6%).

**Fig. 2:**
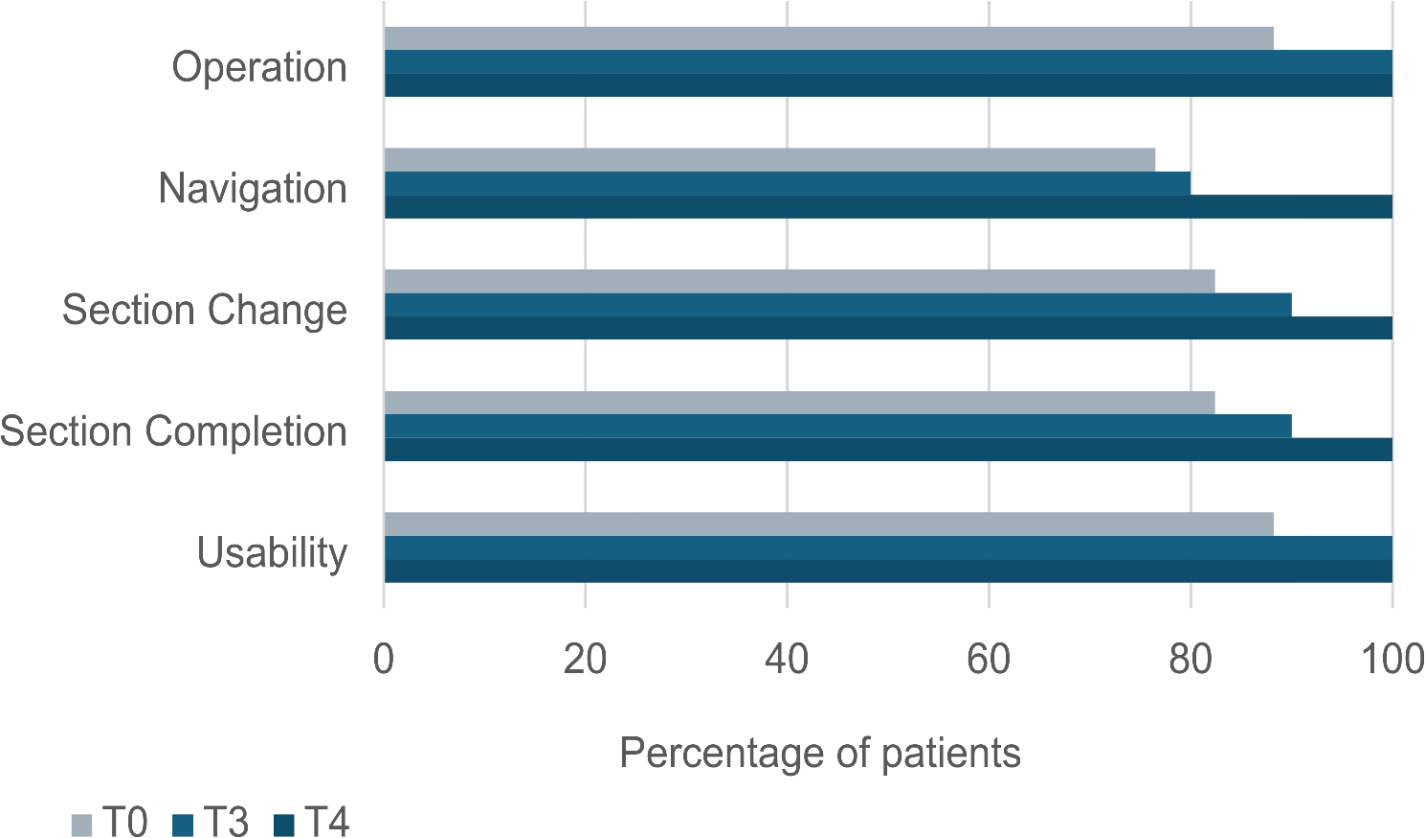
Bar graph of descriptive analysis in the ‘Handling of the digital questionnaire’ section

A significant difference between the paper-based and online-based modalities was determined regarding the completion time of the questionnaire. Patients tended to spend more time completing the online version. Although the completion time of the online-based questionnaire was still longer at T3, after six months, no significant difference was noted. After one year (T4), the comparison of the rated completion time revealed significant results (*p* = 0.032). After the 6-month evaluation, coping with the online version was significantly different between the two groups [14]. A descriptive comparison of the category ‘Handling of the digital questionnaire’ at T0, T3 and T4 is shown in Fig. 2.

### Correlation analysis

In a further correlation analysis, associations between the evaluation results and four selected variables (age, completion time, media usage and IT skills) were examined. There was no association between patient age and media usage.

In reference to the scope range of the questionnaire (paper: *r* = 0.448, *p* = 0.106); online: *r* = 0.393, *p* = 0.308), as well as the suitability (paper: *r* = 0.375, *p* = 0.086) and laboriousness (online: *r* = 0.347, *p* = 0.173) of the interrogation modality, nonsignificant correlations could be observed [14].

In the subsection ‘Handling of the digital questionnaire’, a significant association in the category ‘section completion’ (*r* = 0.714; *p* = 0,001) was found.

**Fig. 3:**
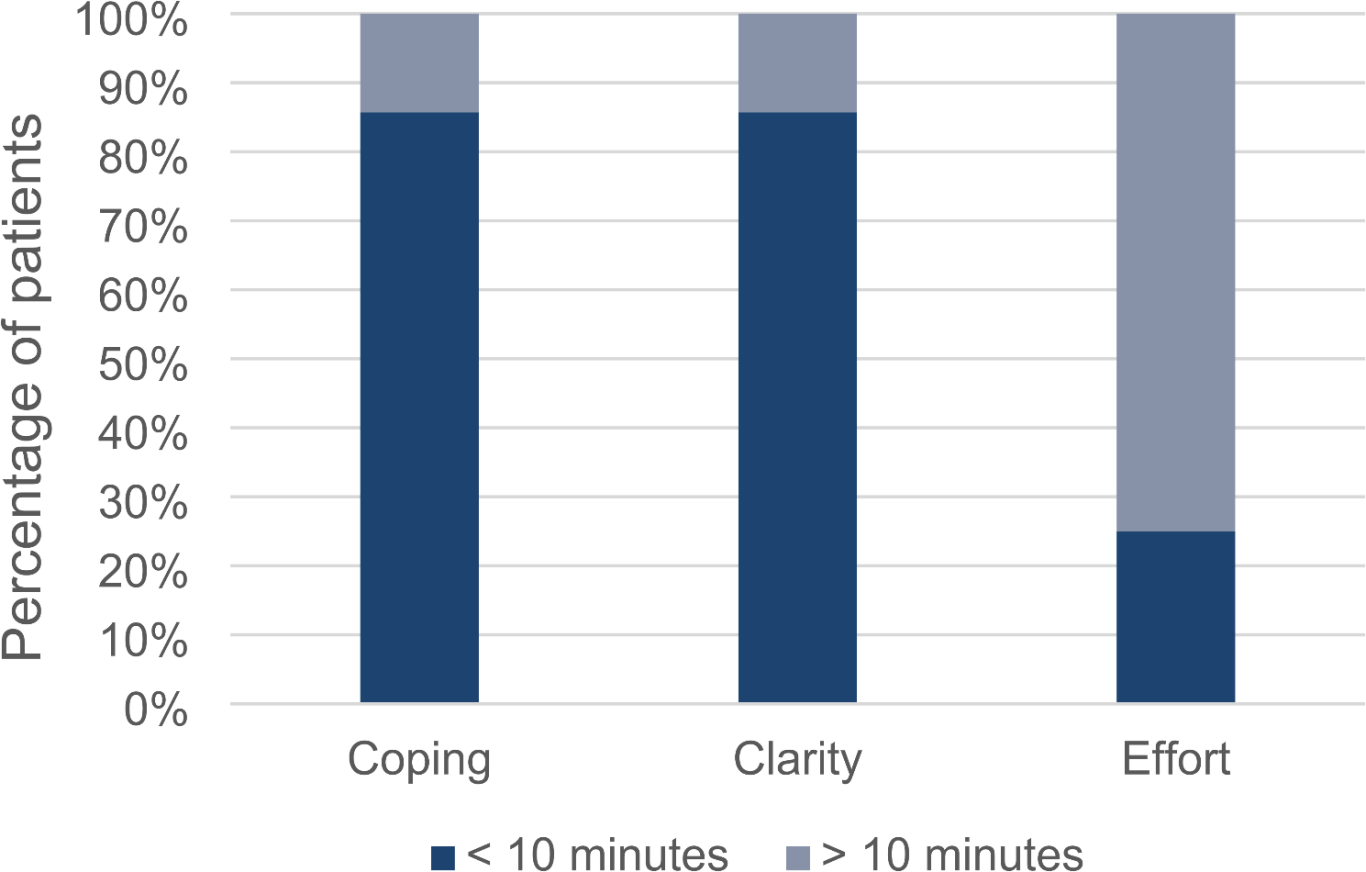
Column graph associating completion time and acceptance variables of the online-based questionnaire at T0

With respect to the variable ‘completion time’ regarding the online-based modality, significant associations were observed in the categories coping (*r* = 0.530; *p* = 0.029), clarity (*r* = 0.530; *p* = 0.029) and effort (*r* = 0.593; *p* = 0.012) [14]. (Fig. 3)

No significant associations between completion time and handling of the digital questionnaire were noted.

Associations regarding the use of technology and other variables were observed in reference to the frequent use of computers or mobile phones, whereas the use of tablets and the internet in general showed no such correlation. As 100% of the individuals stated frequent smartphone usage, the variable was assigned as a constant, and no correlation analysis was possible. Frequent computer use was significantly associated with a positive rating of the variables suitability (*r* = 0.529; *p* = 0.002) and laboriousness (*r* = 0.529; *p* = 0.002) of the paper-based questionnaire. Patients in the online-based study arm showed a significant correlation between computer usage and lower effort (*r* = 0.554; *p* = 0.015). Individuals with subjectively rated high IT skills had a significant correlation with the variable ‘simplified communication’ (*r* = 0.484; *p* = 0.022), which is an eligible reason for the implementation of routinely performed ePRO interrogations. Table 3 shows all the observed associations at T0 with *r* > 0.3.

**Table 3:** Correlation analysis of the evaluation data at T0

		Study group					
Variable	Category	Paper-based (*n* = 23)			Online-based (*n* = 17)		
		Eta (r)	r^2^	*p*	Eta (r)	r^2^	*p*
Age	Scope range	0.448	0.201	0.106	0.393	0.154	0.308
	Suitability	0.375	0.141	0.086	-	-	-
	Laboriousness	-	-	-	0.347	0.12	0.173
	Lack of technical knowledge	-	-	-	0.366	0.134	0.148
	Simplified communication	-	-	-	0.389	0.151	0.123
	Patient-centeredness	0.349	0.122	0.103	-	-	-
	Fewer emergencies	0.305	0.093	0.158	-	-	-
	Faster diagnostics	0.365	0.133	0.087	-	-	-
	Section change	-	-	-	0.364	0.132	0.151
	Section completion	-	-	-	0.714	0.509	**0.001** ^**^
Completion time	Coping	-	-	-	0.53	0.281	**0.029** ^**^
	Clarity	-	-	-	0.53	0.281	**0.029** ^**^
	Effort	-	-	-	0.593	0.352	**0.012** ^**^
	Complexity	-	-	-	0.467	0.218	0.059
	Simplified communication	-	-	-	0.316	0.099	0.216
	Fewer emergencies	-	-	-	0.543	0.295	**0.024** ^**^
	Operation	-	-	-	0.387	0.149	0.264
	Navigation	-	-	-	0.312	0.097	0.307
	Section change	-	-	-	0.337	0.114	0.151

	Category	Kendall tau c	r^2^	*p*	Kendall tau c	r^2^	*p*
Media usage							
Mobile phone	Suitability	0.545	0.297	**0.012** ^**^	-	-	-
	Laboriousness	0.364	0.132	0.149	-	-	-
Computer	Suitability	0.529	0.279	**0.002** ^**^	-	-	-
	Laboriousness	0.529	0.279	**0.002** ^**^	-	-	-
	Coping	-	-	-	0.415	0.172	0.051
	Clarity	-	-	-	0.415	0.172	0.051
	Effort	-	-	-	0.554	0.307	**0.015** ^**^
	Complexity	-	-	-	0.318	0.101	0.25
Tablet	Effort	-	-	-	0.332	0.11	0.237
IT skills	Scope range	-	-	-	0.346	0.119	0.113
	Coping	-	-	-	0.304	0.092	0.121
	Clarity	-	-	-	0.304	0.092	0.121
	Laboriousness	-	-	-	0.429	0.184	0.053
	Simplified communication	-	-	-	0.484	0.234	**0.022** ^**^
	Patient-centeredness	-	-	-	0.388	0.151	0.103
	Section change	-	-	-	0.304	0.092	0.121
	Section completion	-	-	-	0.304	0.092	0.121

Correlation: r > 0.3, ^**^significant (α = 5%), r^2^ = variance, *p* = exact significance

After six months (T3), no significant correlations between age and the evaluation results were found. In contrast, correlations between completion time over ten minutes of the paper version and negative ratings in the categories of suitability (*r* = 0.816; *p* = 0.004) and complexity (*r* = 0.654; *p* = 0.040) were observed. With respect to the completion time of the online version, no significant associations were observed after six months. Table 4 shows all the observed correlations at T3 with *r* > 0.3.

**Table 4:** Correlation analysis of the evaluation data at T3

Variable	Paper-based (*n* = 10)				Online-based (*n* = 10)		
	Category	Eta (r)	r^2^	*p*	Eta (r)	r^2^	*p*
Age	Scope range	0.651	0.424	0.145	-	-	-
	Clarity	-	-	-	0.4	0.16	0.253
	Effort	0.473	0.224	0.167	-	-	-
	Laboriousness	0.366	0.134	0.298	0.4	0.16	0.253
Completion time	Scope range	0.585	0.342	0.231	-	-	^**−**^
	Effort	0.312	0.097	0.379	-	-	^**−**^
	Suitability	0.816	0.666	**0.004** ^**^	-	-	^**−**^
	Complexity	0.654	0.428	**0.040** ^**^	-	-	-
	Laboriousness	0.346	0.119	0.328	-	-	-
	Data privacy	0.564	0.318	0.089	-	-	^**−**^
	General burden	0.387	0.149	0.27	-	-	-
	Patient-centeredness	0.587	0.345	0.074	-	-	-
	Fewer emergencies	0.469	0.219	0.171	-	-	-
	Faster diagnostics	0.436	0.19	0.208	-	-	-
	Improved therapeutic concepts	0.387	0.149	0.27			

Correlation: r > 0.3, ^**^significant (α = 5%), r^2^ = variance, *p* = exact significance

## Discussion

The primary objective of this study was the analysis of an electronic patient-reported outcome assessment regarding health-related quality of life in patients with endometriosis. A comparison of the two interrogation modalities, paper-based and online-based, for the evaluation of acceptance and usability was performed.

A low rate of return concerning the evaluation forms was observed in both study arms (T0: paper: 41.1%, online: 30.4%; T3: *n* = 17.9%). Possible reasons could be additional effort, loss of documents or loss of contact caused by the long follow-up period, as well as a decreased sense of responsibility due to anonymity. For this reason, external validation of the results is limited, although important first tendencies regarding the acceptance and usability of the online tool CANKADO were shown. Concerning the high dropout rate, a study by Judson et al. reported a decrease in compliance after 16 weeks, with possible explanations such as neglect, lack of time, and technical or health-related burdens mentioned by the patients [19]. More than 80% of the individuals rated the HRQoL assessment via the EHP-30 questionnaire as customized to their needs. While the scope range of the questionnaire was primarily rated as adequate by patients of both interrogation modalities, a more positive result was detected with respect to the online version.

Bourdel et al. previously postulated the extent of the classic paper-based EHP-30, leading to a completion time of 10–15 min on average. This could be seen as a time-consuming factor and therefore impedes routine implementation in the clinical setting [20].

A different study investigating an electronically performed psycho-oncological interrogation in patients with breast cancer reported coherent results with our investigation. According to the authors, an electronically based interrogation was better accepted and could be seen as a less time-consuming alternative [21]. Although the detected values were categorized at a lower end of the range, our study revealed a significantly longer self-assessed completion time for the online-based questionnaire. As completion of the online version was conducted at the patients’ home, after adequate registration to the online application CANKADO, it is difficult to investigate possible explanations for the enormous difference between the valuables. On the one hand, subjective causes such as a lack of technical knowledge or low media usage in daily life could be considered possible explanations, especially with respect to the significant difference in self-assessed IT skills between the two interrogation modalities. Thus, Table 2 shows a significantly greater number of patients with low IT skills (*p* = 0.026) in the online-based group. Furthermore, another focus should be on the long follow-up period of several months between the interrogations and the consequent absence of customization to the online application. A higher frequency of application usage could have led to more experienced handling of the online tool with a shorter completion time.

Small yet nonsignificant differences between the two interrogation modalities could be observed in reference to the category’s complexity and laboriousness. In contrast to our findings, a study by Hartkopf et al., who investigated ePRO assessment in breast cancer patients, reported more positive results in the rating of their applied online tool within the categories of suitability, laboriousness and complexity. In line with our investigation, data privacy was the primary counter reason for a routinely performed ePRO assessment in a clinical setting [22].

Reasons for the improvement of care by an applied ePRO interrogation included simplified communication, faster diagnostics and improved therapeutic concepts by the main percentage of our patients. In particular, improvements in doctor–patient communication through the use of tablet-based symptom surveillance were observed in earlier studies. Their findings indicated greater patient satisfaction when interest and demand for information were expressed by the practitioner [23].

Cowan et al. reported, in their postoperative ePRO assessment in gyno-oncological patients, high feasibility and acceptance from the patient’s side. However, it seems crucial to include both patients and practitioners in the development process. Similarly, sufficient reflection of patients’ symptomatology, as well as high usability for clinical staff, can be ensured, and additional burden in daily clinical workflows can be prevented. Additionally, by offering easy electronic access to symptom reports for clinical providers prior to appointments, postinterventional consultations could become more efficient [24]. Other studies emphasize that by performing regular ePRO assessments, evaluations of the impact, appropriateness, quality, and performance of health care in patients with endometriosis can be facilitated. Different therapy regimens can be compared on the basis of ePRO results and adjusted in the frame of shared decision-making. In addition, comparisons of best practices between different clinicians in endometriosis care on the basis of ePROs would be possible [25]. Other developed ePRO assessment tools, such as the Endometriosis Symptom Diary (ESD) or Endometriosis Impact Scale (EIS), which were investigated in a study by Gater et al., also showed promising results in the field of endometriosis. One of the principal arguments for an electronic PRO assessment is the guarantee of data loss. The compliance rate was high, and the completion time was appropriate. Further studies are needed to analyze and define score values and their interpretation [26].

Previous research regarding medical misinformation on social media platforms such as Instagram and TikTok revealed immense differences concerning content reliability and the provision of evidence-based information [27–29]. For this reason, the importance of a reliable tool for monitoring guidance and therapeutic effects in patients with endometriosis seems to be evident. By routinely maintaining digital contact with professional healthcare providers, patients with endometriosis can gain more trust in medical information and advice, leading to increased therapy adherence and thereby improved HRQoL.

As shown in Fig. 2, the performance of the digital questionnaire was rated very positively by our patients at 0, 6 and 12 months of evaluation. Technical problems mentioned throughout the follow-up period referred to issues concerning unclear presentation, item selection, loading difficulties and memory function. In the beginning, patients complained about difficult access to the online questionnaire, in addition to memory issues. Patients reported that if they were interrupted while completing the questionnaire, they had to complete it from the beginning instead of continuing with the current question because of the deletion of previous answers. This might have led to an enormous increase in completion time and is one possible explanation for the investigated difference between the two modalities. It is obvious that further improvement regarding a user-friendly surface, appropriate memory function and the possibility of navigation to the current question after having paused the interrogation is indispensable to guarantee high usability and acceptance of the online application. In cooperation with the IT support of CANKADO, most issues had already been resolved during the interrogation period, probably leading to more positive results concerning the handling of the digital questionnaire at T3 and T4. In addition, patients were asked to provide constructive feedback and suggestions, e.g., the integration of a menstrual cycle calendar, with the aim of future improvement of the online application.

While several low to moderate yet nonsignificant associations between age and evaluation data were observed in our findings, a former study by Drewes et al. reported increased acceptance of internet-based side effect monitoring for younger patients. However, the age range in the mentioned study was 28–76 years, which was wider than that in our study, which was 17–52 years. In addition, the category ‘younger patients’ was defined as those under 56 years of age [30]. As our patients’ mean age was 29.52 years (SD: 7.89), it seems logical that few significant associations were observed regarding the entire young patient population.

In contrast, completion time had a potentially significant effect on the acceptance and usability of both interrogation modalities. As depicted in Fig. 3, significant moderate correlations were observed regarding the categories coping (*p* = 0.029), clarity (*p* = 0.029) and effort (*p* = 0.012) and completion time over ten minutes of the online-based questionnaire. Additionally, at T3, a longer completion time was significantly associated with poorer suitability (*p* = 0.004) and greater complexity (*p* = 0.040) of the paper-based modality. In reference to our results, Jones et al. proposed the application of the short-form questionnaire EHP-5 for clinical use in one of their recent studies [6]. This short version of the EHP-30 has been applied in previous research and was already integrated into the Endo-App, an online application designed in Germany for the improvement of self-management in patients with endometriosis [11, 31]. In contrast to the 53 questions of the EHP-30, only 11 questions are included in the EHP-5. Despite a certain loss of information involving the shortage of items, a considerable decrease in completion time could be obtained.

With respect to media usage and IT skills, our results indicated a possible significant correlation between the frequent usage of a computer and less effort concerning the online-based modality. Those individuals reporting high IT skills seemed to have chosen the option ‘simplified communication’ as the reason for improvement in care more frequently. In an earlier study on ePRO assessment in gyno-oncological patients, researchers reported correlated results. Thus, individuals with frequent use of computers, tablets or the internet in general and who additionally self-assessed high IT skills rated a routinely performed ePRO assessment as more suitable, less difficult and less effortful [22]. Therefore, the assumption can be made that frequent media usage and higher IT skills can be associated with higher acceptance and usability ratings of online-based interrogations.

### Limitations and strengths of the study

There are several limitations of the current study. First, both factors, the single-center study design and the subsequent low postal return rate, led to a small sample size. Therefore, external validation of the observed results is limited and should be interpreted according to the investigative circumstances. Furthermore, despite the use of double-blind randomization, with the aim of equivalent participants’ distribution, a certain selection bias may have occurred.

Second, the large follow-up period between the individual evaluations at 0, 6 and 12 months could have caused a certain loss-to-follow-up bias, which should be considered in the interpretation of the data.

Third, the exclusive application of the German version of the EHP-30 questionnaire and evaluation forms, owing to the German user interface of the online application, may have decreased the number of participants during patient recruitment. In addition, it must be considered that patients were free to use the online application on their computer, tablet or smartphone. Consequently, no further correlations between the applied digital device and the user experience were investigated. This could be an interesting outcome for further studies.

Owing to our frequent assessments of the EHP-30 and the evaluation forms at multiple points in time within a period of one year, a great gain of data was obtained. This could be valuable data collection for follow-up studies. In addition to the observed small sample size, certain tendencies toward factors influencing the acceptance and usability of an ePRO assessment could be observed. Thus, a completion time of less than ten minutes could be crucial, and therefore, the application of the short-form questionnaire EHP-5 would be an eligible solution.

## Conclusions

The study identified the online application CANKADO as a suitable and well-accepted ePRO assessment tool for measuring HRQoL in patients with endometriosis. Patients showed great interest in routinely performing ePRO assessments in the clinical context and saw improvements concerning simplified doctor–patient communication, faster diagnostics and improved therapeutic concepts. The acknowledgment of the strong negative impact of chronic gynecological conditions such as endometriosis on patients’ HRQoL shows the relevance of assessing this parameter for evaluating patients’ health status development. A routinely performed ePRO assessment could therefore help practitioners and patients keep track of actual symptom development, especially after beginning or changing the therapeutic regimen. The effects of different therapies on HRQoL in individuals could be collected via ePRO assessment and evaluated in future studies to create the opportunity for a more personalized therapeutic approach in patients with endometriosis. Through the implementation of CANKADO in hospital information systems, simplification of direct symptom and therapy monitoring and thereby improvement of doctor–patient communication could be achieved. In summary, these improvements may lead to increased patient and practitioner satisfaction. While age and media usage seemed to have small associations with a positive user experience, an appropriate completion time of less than ten minutes was observed as a principal factor. Further prospective studies with larger samples are needed to confirm these findings.

## Data Availability

The dataset used and/or analyzed during the current study are available from the corresponding author on reasonable request.

## Abbreviations

DIGA: Digitale Gesundheitsanwendung
EHP-30: Endometriosis Health Profile-30
ePRO: Electronic Patient-Reported Outcome
HRQoL: Health-Related Quality of Life
LMU: Ludwig-Maximilians-Universität
PRO: Patient-reported outcome
